# Effectiveness of Emotion Regulation Group Therapy on Craving, Emotion Problems, and Marital Satisfaction in Patients with Substance Use Disorders: A Randomized Clinical Trial

**Published:** 2019-10

**Authors:** Fatemeh Zargar, Nasim Bagheri, Mohammad Javad Tarrahi, Mehrdad Salehi

**Affiliations:** 1 Department of Health Psychology, School of Medicine, Isfahan University of Medical Sciences, Isfahan, Iran.; 2 Department of Clinical Psychology, Isfahan (Khorasgan) Branch, Islamic Azad University, Isfahan, Iran.; 3 Department of Epidemiology and Biostatistics & Behavioral Sciences Research Center, School of Medicine, Isfahan University of Medical Sciences, Isfahan, Iran.; 4 Department of Psychiatry, School of Medicine, Isfahan University of Medical Sciences, Isfahan, Iran.

**Keywords:** *Craving*, *Emotion*, *Marital Relationship*, *Substance Use Disorder*

## Abstract

**Objective:** Psychological and environmental factors, such as difficulties in emotion regulation (ER) and marital problems, are involved in relapse and craving in patients with substance use disorders. Emotional regulation therapy can help maintain opioid withdrawal and improve marital relations by focusing on appropriate adjustment of emotions. This study aimed to evaluate the effectiveness of emotion regulation therapy on craving, emotion regulation, and marital satisfaction in patients with substance use disorders.

**Method** **:** This randomized controlled clinical trial with pretest and posttest was performed in 2014 in Noor hospital, Isfahan, Iran. In this study, 30 patients who were admitted to the addiction center of Noor hospital were selected using purposive sampling. They were assigned into two groups randomly: (1) 15 patients in treatment as usual (TAU group); (2) 15 patients in emotion regulation group therapy (ERGT). The ERGT group received eight weekly treatments, based on Gross model, to learn recognize emotions and their effects, overcome obstacles of positive emotions, accept emotions, identify regulatory maladaptive and adaptive strategies of emotions, and modify behavior. Before and after the emotion regulation sessions in experimental group, Craving Beliefs Questionnaire (CBQ), Emotion Regulation Questionnaire, and Dyadic Adjustment Scale (DAS) were administered in both groups.

**Results: **The results of analysis of variance indicated that mean scores of marital adjustment increased in ERGT (93.66 ± 15.81) compared to TAU group (55.26 ± 20.98) and the mean scores of craving were decreased in ERGT compared to TAU group (56.66 ± 18.39 and 105.2 ± 34.5, respectively). Also, most aspects of ER improved in ERGT compared to TAU group, and the total score of ER was increased in ERGT significantly (96.69 ± 5.38 in ERGT versus 73.70 ± 5.05 in TAU).

**Conclusion: **Based on the findings of this study, emotion regulation group therapy has a significant effect on reducing Craving and improving marital adjustment and emotion regulation in Patients with Substance Use Disorders. So, it can use as a useful psychotherapy in addiction treatment centers.

Substance use disorder (SUD) includes a cluster of cognitive, behavioral, and physiological symptoms, indicating that the individual continues using the substance despite significant substance-related problems (1). In Iran, 20% of 15-60-year-olds have issues with drugs. 

Over the past decade, increasing attention has been paid to difficulties in emotion regulation that cause many psychological problems such as substance use disorders (SUDs) (2, 3). Emotion regulation (ER) can be defined as the mechanism through which people regulate their emotions to achieve desired results (4).

Based on the Gross model (5), a low emotion regulation level due to an inability to confront and manage emotions effectively plays a role in substance abuse onset. The Gross ER process model consists of a series of adaptive and maladaptive strategies. Cognitive reappraisal, which focuses on the long-term negative consequences associated with substance use, and problem-solving are categorized as adaptive strategies (6). Avoidance and suppression are conceptualized as maladaptive strategies (4). Various studies have shown cognitive reappraisal strategies are superior to other strategies (7-9). These ER skills are superior to suppression in SUDs, but some studies that examined the relationship between emotion regulation strategies and psychopathology showed that the relationship between reappraisal and substance use was small (4, 10). 

 In Gross model of ER, participants were trained to recognize their emotions, arousing situations, and short-term and long-term effects of emotions. Also, they learned to overcome obstacles of positive emotions, accept emotions, and identify regulatory maladaptive and adaptive strategies of emotions such as avoidance, problem-solving, and reappraisal. Also, they learned emotion expression and behavior modification through environmental reinforcement (11). 

Emotion regulation and distress tolerance are needed for successful opioid withdrawal and craving resistance (11). Difficulties in emotion regulation (especially negative emotions; NE) are important stimulus for craving and substance use. Cigarette smokers and alcohol users reported that the main reason for their tendency to smoke and drink alcohol was negative affection (12, 13). In contrast, abusers who used more adaptive strategies of emotion regulation were more successful in treatment courses (14). Studies have shown that craving and negative affection are associated with substance withdrawal and can predict future relapse (15, 16). 

Several studies have found that treatment with essential components of ER therapy can reduce craving in patients with SUDs (4, 17-21). One study showed significant reduction in anger symptoms of drug-dependent individuals after emotion regulation training based on Gross model (22). However, a few studies examined Gross's structured protocol of ER to reduce craving in SUDs.

On the other hand, ER is an essential component for successful interpersonal relationships (23), especially in marital relationship, which is a protective factor against relapse and an important predictor of treatment outcome in SUDs. One of the problems in patients with SUD is marital dissatisfaction, and spouses who have the tendency to use substances, have low marital satisfaction (24). Generally, couples with emotional instability and impulsivity are considered undesirable partners, while those with suitable ER mechanisms have happier relationships (25). Studies have shown that ER training can reduce impulsivity, which is the source of aggression and conflict between couples (26). Also, ER training improves marital satisfaction and intimacy between couples in various groups (27-29). Therefore, ER in people with SUDs is important for improving relationships and treatment outcomes.

Although some studies have shown the effectiveness of ER therapy on craving in people with SUDs and marital satisfaction, no study has examined the effectiveness of ERGT on craving and marital satisfaction of people with SUDs simultaneously. 

In this study, the effectiveness of emotion regulation therapy was examined on craving, emotion dysregulation, and marital adjustment in patients with substance use disorders based on Gross's model. This study hypothesized that the experimental group gained improvements in ER scores using ERGT compared to the TAU group. Moreover, it is assumed that ERGT reduces craving and marital dissatisfaction in ERGT group, compared to the TAU group.

## Materials and Methods

***Participants and Procedure***

This randomized controlled clinical trial was conducted on all male patients aged 20-50 years with SUDs who admitted to the addiction center of Noor hospital, Isfahan, Iran, from November to June 2014 (IRCT NO: 201505157227N2). 

The sample size of the study was determined at 15 patients for each group based on a similar study (18) with an error type I (α = 0.05), error type II (0.2), an effect size of 16, and standard deviation of 17 and 14. After reviewing the files of patients with SUDs, 40 patients who met the inclusion criteria were selected using purposive sampling. Subsequently, the patients were invited to participate in the study and were assigned into experimental (ERGT) and control groups using a random number table. 

The inclusion criteria were: (1) diagnosis of SUDs according to Diagnostic and Statistical Manual of Mental Disorders-5 by a psychiatrist, (2) subjected to no psychological treatment for at least a month before the study, (3) minimum and maximum ages of 18 and 60 years, respectively, (4) minimum third level of education in middle school, (5) willingness to participate in the study, (6) lack of psychotic symptoms, and (7) the presence of other psychiatric disorders, such as mood disorders and suicidal thoughts. On the other hand, the participants who missed more than two therapeutic sessions and reused substances were excluded from the study. 

In addition to the usual treatments for patients with SUDs (eg, methadone therapy), the experimental group was subjected to 8 sessions of ERGT on a weekly basis according to the protocol developed by Gross et al (5, 30). 

The outline of the protocol is shown in Table 1.

Both ERGT and control groups were asked to respond to the items of the instruments employed in this study at pretest and posttest in the addiction center of Noor hospital. These tools included Difficulties in Emotion Regulation Scale (DERS), Dyadic Adjustment Scale (DAS), and Craving Beliefs Questionnaire (CBQ). Moreover, the demographic characteristics of the respondents were obtained at baseline. All the ERGT sessions were held in Noor hospital and administered by a trained psychologist. Each session lasted 120 minutes from 10 AM to 12 PM on Wednesdays. If any participants missed 1 or 2 sessions, they had to attend additional sessions. After finishing the study, the patients in the control group who were interested in psychotherapy were invited to attend ERGT sessions similar to those that were held for the experimental group. 

**Figure 1:**
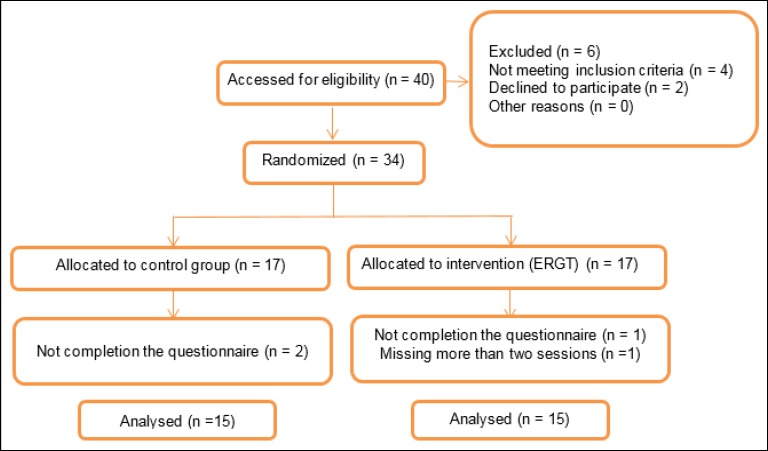
The CONSORT Flow Diagram Indicative of the Progress of All Participants

Figure 1 presents the consolidated standards of reporting trials (CONSORT) flow diagram of the study.

Overall, the t test and chi-squared test were used to compare significant differences between mean ages and other demographic variables, such as marital status and educational level in the two groups, respectively. Moreover, the Multiple Analysis of Covariance (MANCOVA) was used to compare ERGT and TAU groups regarding ER, craving, and marital adjustment. 

***Questionnaires***

***Difficulties in Emotion Regulation Scale***

The DERS is a brief, 36-item, self-report questionnaire designed to assess multiple aspects of emotional regulation (31). The items are scored based on a 5-point Likert scale. The reverse-scored items are 1, 2, 6, 7, 8, 10, 17, 20, 22, 24, and 34, with higher scores representing greater problems with ER. This scale includes 6 subscales: (1) non-acceptance of emotional responses (NONACCEPT), measuring the tendency to react to a negative emotion (NE) with a secondary NE, such as shame and guilt; (2) difficulties engaging in goal-directed behaviors (GOALS), measuring the ability to engage in goal-directed behaviors while experiencing NE; (3) impulse control difficulties (IMPULSE), measuring the ability to refrain from acting impulsively when experiencing NE; (4) lack of emotional awareness (AWARE), measuring the tendency to attend emotional states; (5) limited access to emotion regulation strategies (STRATEGIES), measuring the belief that little can be done to effectively regulate emotions; and (6) lack of emotional clarity (CLARITY), measuring the ability to clearly identify emotional states. 

This scale obtained a high internal consistency (α = 0.93); moreover, Cronbach's alpha for 6 subscales of this questionnaire was estimated at 0.77, 0.71, 0.83, 0.49, 0.84, and 0.52, respectively (31). Furthermore, the content validity of the Persian version of the scale was confirmed by psychology experts, and the reliability of the Persian version of the scale based on the alpha coefficient was determined at 0.91(32). In addition, five clinical psychologists confirmed the formal validity of the test. The reliability of the DERS was obtained at 0.90 in this study.

***Dyadic Adjustment Scale***

The DAS (33) is a 32-item scale widely utilized for measuring relationship adjustment in married couples or similar dyads. Original factor analysis of the DAS identified four subscales, namely Dyadic Consensus, Dyadic Satisfaction, Dyadic Cohesion, and Affection Expression (33). This tool showed high content validity, and the correlation between DAS and Locke- Wallace marital adjustment test was estimated at 0.86 and 0.88 in married and divorced respondents, respectively (33). Concurrent validities of DAS and Enrich Scale were 0.84 and 0.81 in male and female respondents, respectively. This scale has been reported to have high reliability in Iranian male (0.89) and female subjects (0.91) (34, 35).

***Craving Beliefs Questionnaire***

This 20-items questionnaire is a self-report scale measuring the beliefs about craving for drug use considering mental, physical, and behavioral aspects. It is scored based on a 7-point Likert scale from 1 (strongly disagree) to 7 (strongly agree). One study showed the questionnaire had suitable validity and reliability (36). The reliability of the Persian version of CBQ using Cronbach's alpha was estimated at 77.0 (37). Moreover, the concurrent validity of the CBQ was investigated using the correlation between craving intensity and attentional bias for drug-related stimuli (38). 

***Ethical Considerations***

Written informed consent was obtained from all participants, and they were all informed of the research procedure, objectives, evaluation, and confidentiality of their data and names since each participant was given a numerical code. However, the program coordinator had access to the information provided by the participants. The participants had the right to withdraw from the study at any time.

## Results

Table 2 summarizes the demographic characteristics of the participants. According to the results, there were no significant differences between the two groups regarding demographic characteristics. 

The hypothesis of the study was checked using MANCOVA. The results of Kolmogorov-Smirnov test regarding the normal distribution of the data showed same distribution in the samples of the two groups. Levene’s test results confirmed the null hypothesis about the equality of error variance of the dependent variables (F = 0.225, P = 0.639). Furthermore, Box’s M Test of Equality of Covariance Matrices showed that the observed covariance matrices of the dependent variables were equal across groups (Box’s M = 5.97, F = 1.84, P = 0.138). 

Observed power in Table 3 shows that sample size is sufficient for all variables expect for craving and STRATEGIES subscale of ER scale. 

The mean scores of dependent variables (eg, craving, marital adjustment, and ER) in the pretest were imported into MONCOVA model as covariate variables (Table 3). According to Table 3, there was a significant decrease in mean values of all subscales of the DERS in the ERGT group except "STRATEGIES" and "AWARE" subscales at posttest, compared to the control group. However, no significant difference was observed between pre- and posttest scores of the ERGT group in AWARE subscale; nonetheless, it was close to 0.05. 

In addition, Table 3 reveals that ERT can reduce craving in patients with SUDs, compared to the control group. Furthermore, there was an increase in the marital satisfaction level of ERGT group, meaning that ERGT can help participants express more affection towards their wives. Additionally, they have found greater agreement on a variety of issues, such as managing family finances or making important decisions, and doing more collaborative activities. Consequently, all hypotheses were confirmed in this study. These significant differences between pre- and posttest results can be attributed to the increase in ER scores, especially due to identification and acceptance of NEs (eg, CLARITY and NONACCEPT subscales of DERS scale), reduction of reactive responses to NE (eg, IMPULSE), and engagement in goal-directed behaviors despite NE (eg, GOALS). 

**Table 1:** Outline of the Emotion Regulation Therapy According to Gross et al

**Session**	**Session contents**
1	Introducing participants to one another Introducing the rules of the group Introducing the types of emotions (eg, positive and negative) Introducing craving as a negative internal event (eg, negative emotions)
2	Recognizing the situations that arouse emotions Discussing issues regarding different aspects of emotion and outcomes of emotions (eg, short- and long-term)
3	Monitoring and recognizing one’s own emotional experiences Introducing regulatory strategies of emotions (eg, avoidance, reappraisal) Identifying the strategies of emotions in each participant
4	Teaching the strategies to prevent social isolation and avoidance as well as improve problem-solving ability Introducing interpersonal and marital conflict solving (eg, active listening, correct emotion expression)
5	Teaching the strategies to manage obsessive thinking and worrying
6	Introducing the effect of emotional states Teaching the reappraisal strategy
7	Identifying the methods each person applies to inhibit the emotions and their outcomes Preventing emotional inhibition using confrontation and behavior modification
8	Reviewing the contents thought in each session Assessing the ability of each participant considering the achievement of therapy objectives

**Table 2:** Demographic Characteristics of Patients in ERGT and TAU Groups

**Variable**		**ERGT**	**TAU**	**P value**
Age		5.77±25.70	2.39 ± 24.85	0.54**
Education Level				0.88***
High School		10 (25)	11 (27.5)	
Bachelor		10 (25)	9 (22.5)	
Marital Status				0.99***
Married		10 (25)	10 (25)	
Single		10 (25)	10 (25)	

Abbreviations: ERGT, emotion regulation group therapy; TAU, treatment as usual.

Data are presented as mean ± SD or No. (%).

** t test

*** Chi- square test

**Table 3:** Means, Standard Deviations, and Comparison of Outcome Measures (Craving, Marital Adjustment, and Emotion Regulation) at Post Treatment in the 2 Groups

**Outcome Measure**	**Pretest**	**Posttest**		**F**	**P ** **Value**	**Eta squared**	**Observed power**
	**Mean ± SD**	**Mean ± SD**					
Craving							
ERGT	116.6± 16.4	56.66±18.39		21.883	0.001	0.448	0.448
TAU	119.1±11.64	105.2± 34.5					
Marital adjustment							
ERGT	55.26 ± 16.15	93.66 ± 15.81		25.685	0.001	0.488	0.998
TAU	57.26 ± 16.22	55.26 ± 20.98					
Emotion dysregulation							
NONACCEPT	ERGT	21.93±2.60	21.33± 3.77	13.29	0.001	0.464	0.996
	TAU	17.33±3.94	11.73± 2.91				
GOALS	ERGT	15.60± 1.76	14.33± 1.58	24.81	0.001	0.592	0.994
	TAU	13.93± 3.26	10.73± 2.65				
IMPULSE	ERGT	20.00± 3.20	18.46± 3.02	15.12	0.001	0.750	1.000
	TAU	21.06± 2.86	15.13± 2.13				
CLARITY	ERGT	7.60± 2.09	7.40± 1.76	24.73	0.001	0.718	1.000
	TAU	8.26± 2.46	4.93± 1.86				
AWARE	ERGT	19.60± 3.41	17.80± 2.93	3.88	0.06	0.802	1.000
	TAU	17.40± 2.50	14.60± 2.91				
STRATEGIES	ERGT	18.80± 3.91	16.80± 3.23	0.734	0.399	0.000	0.050
	TAU	19.00± 3.25	16.06± 3.19				
Emotion regulation (Total score)	ERGT	104.12±9.21	96.69±5.38	113.079	0.001	0.807	1.000
	TAU	97.65±5.62	73.70±5.05				

Abbreviations: ERGT, Emotion Regulation Group Therapy; TAU, Treatment as Usual.

## Discussion

This study showed that ERGT could reduce difficulties in ER among patients with SUDs, compared to TAU. Several studies have highlighted the role of ER deficits in the formation of substance-related disorders (3, 39, 40). The present study revealed that ERGT could reduce difficulties in all subscales of DERS, except STRATEGIES and AWARE, which means that the participants learned to identify their NEs (anxiety and anger), accept and respond to NEs without secondary NEs (shame, anger, and guilt), and engage in goal-directed behaviors while experiencing NEs, and refrain from acting impulsively when experiencing NEs. However, they still had low ability to manage emotional states and modify them. 

The lack of an increase in scores of STRATEGIES subscale can be explained by short-term duration of the present study (eg, 8 weeks). Perhaps in the long-term, the participants can gain higher STRATEGIES subscale scores by mastering on applying ER strategies, such as reappraisal and problem-solving. On the contrary, other studies showed increasing ER skills during this time. It has been shown in a study that cocaine-dependent and alcohol-dependent patients reported significant difficulties in emotional awareness and impulse control during the first week of treatment, compared to the control group (2, 3). However, significant improvements were observed during the treatment regarding awareness and clarity of emotion (3). 

Secondly, it seems that ER training can reduce difficulties in ER and craving via reducing impulsivity (eg, higher scores in IMPULSE subscale). The skills, such as identifying emotions and their acceptance, as well as engaging in goal-directed behaviors can reduce impulsivity. When impulse control is improved, the patients could not act based on immediate craving; on the other hand, more long-term training and practice are required for applying strategies that reduce problematic emotions. 

Furthermore, lower scores in STRATEGIES subscale may be indicative of a fear of excessive emotions. Patients with SUDs overestimate the intensity and duration of their emotions; moreover, they have negative evaluations about their ability to manage their NEs (eg, "When I’m upset, I believe that I will remain that way for a long time" or "When I’m upset, I start to feel very bad about myself). These two evaluation biases need to be modified over time which required repeated successful training. 

The results obtained from this study showed that ERGT could reduce craving in patients with SUDs, compared to the TAU. These results are consistent with the findings of previous studies that investigated the effect of ERT on reducing craving in patients with SUDs (4, 17-21). 

Craving is stimulated by exposure to situations where the individuals have previously used drugs. Therefore, learning the strategies to manage one's thoughts and emotions under such circumstances largely reduces the risk of return to substance abuse. In addition, difficulties in ER, especially NEs, are important stimuli for craving and substance use in cigarette smokers and alcohol users (12, 13). Accordingly, the improvement of ER skills through ERGT can reduce craving in patients with SUDs. 

This finding is consistent with the results of studies that showed that reappraisal skills for ER are superior to suppression in the Gross model (7-9), and it is inconsistent with those of other studies in which they revealed insignificant relationship between reappraisal and substance use (4, 10). This inconsistency can be attributed to the materials of the protocol employed in this study which focused on acceptance and reappraisal.

When people learn to reappraise the unpleasant events, they experience fewer NEs (eg, anger, sadness, anxiety, and guilt); therefore, they do not need to use substance for reducing these emotions. This ability is also useful in situations in which individuals attempt to manage craving. In this protocol, participants learned to identify and accept their craving, NEs, and pleasures. They also learned to not act impulsively. Lower tolerance in these individuals force them to find a quick way to get rid of emotions, and substance use is a faster choice related to their condition (41).

Additionally, the involvement in structured goal-directed activities is another important factor in reducing craving that replaces substance-seeking behaviors and was emphasized in the present study protocol. The current study showed that ERGT could improve marital adjustment in patients with SUDs, and there was a significant relationship between ER and marital satisfaction (42). Some studies have revealed that husbands’ ER is most important for marital satisfaction. The males have been proposed to be particularly sensitive to stress in marriage because of their lower tolerance for prolonged negative emotional states (43). 

In the present study, ERGT allows males with SUDs to have more marital satisfaction by managing their negative states, craving, and relapse. In contrast, other studies found an insignificant relationship between the regulation of husbands’ negative emotion and either spouse’s current or future levels of marital satisfaction (23). Although wives’ marital satisfaction of the patients with SUDs was not investigated in this study, given the nature of the disorder, it seems logical that most of the emotional dysregulation belongs to the male patients (eg, husbands). 

The improvement in ER of participants in ERGT group could promote marital adjustment, because they have learned how to express more affection towards their wives. The identification of one's emotions depends on their ability to share their emotions with their spouses. This ability can also help identify their partner’s emotions, thereby improving their relationship. In the same line, ER practices can prevent impulsive and reactive behaviors in response to NEs, which leads to relationship enhancement. Similarly, impulse control helps the participants have suitable negotiation with each other, and they can find greater agreement on a variety of issues, such as managing family finances, making important decisions, and having more collaborative activities. 

The strength of this study was the evaluation of the effectiveness of simple and new psychotherapy on one of the main consequences of substance use (eg, marital satisfaction) and the most important factor involved in the etiology of the SUDs (eg, emotion dysregulation). Therefore, the health care professionals, including psychiatrist, psychologists, and nurses, as well as the patients will be able to use this treatment. 

## Limitation

The main limitation of this study was the lack of comparison between ERGT and the gold standard psychotherapies, such as cognitive behavior therapy. Moreover, the utilization of the male samples and the lack of follow-ups were among other limitations of this study. 

## Conclusion

This study confirmed the hypothesis that patients suffering from SUDs can improve their ER skills and marital satisfaction and deal with their craving by participating in ERGT sessions. Also, the findings revealed that ER skills training can be used as a major component of psychotherapies for SUDs. Moreover, given that emotional dysregulation is a predisposing factor to the SUDs, ER skills can be used in preventive programs.
